# Renal Cell Carcinoma Associated with Mycosis Fungoides: A Paraneoplastic Syndrome

**DOI:** 10.1155/2020/8897183

**Published:** 2020-10-29

**Authors:** Jessica Tran, Auris Huen, Madeleine Duvic

**Affiliations:** ^1^Department of Dermatology, The University of Texas MD Anderson Cancer Center, Houston, TX, USA; ^2^Baylor College of Medicine, Houston, TX, USA

## Abstract

Patients with mycosis fungoides have an increased risk for additional malignancies, particularly hematologic malignancies. Of the malignancies that have been associated with mycosis fungoides, renal cell carcinoma and other solid tumor malignancies have not been studied extensively. In this case series, we describe three mycosis fungoides patients who were diagnosed with clear cell renal cell carcinoma and discuss the potential pathophysiology underlying this association.

## 1. Introduction

Patients with mycosis fungoides (MF) have an increased risk for additional malignancies, particularly hematologic malignancies [1–3]. MF patients are also at an increased risk for solid tumor malignancies, but this association is less frequent and not well-characterized [2]. In 2019, Goyal et al. detailed a link between MF and solid tumor malignancies such as RCC. The study found elevated standardized incidence ratios (SIRs) for kidney and renal pelvis cancer in patients with MF [2]. SIR for kidney and renal pelvis cancer within a year of diagnosis of MF was 12.05. In our study of 672 patients with cutaneous T-cell lymphoma (CTCL) in 2008, 112 patients (17%) had at least one additional malignancy. Overall, patients with CTCL had a 1.79-fold risk for developing an additional malignancy [4]. In this case series, we describe three MF patients who were diagnosed with clear cell RCC and discuss the potential pathophysiology underlying this association.

## 2. Case Presentation

### 2.1. Case 1: Patient A

A 54-year-old male presented to MD Anderson Cancer Center (MDACC) in October 2003 for the management of newly diagnosed stage IA MF. The patient first noted a small brown macule on his left waist in May 2003. Over the next few months, several additional lesions erupted on his left lower abdomen, back, and neck. He also experienced abdominal bloating. His local dermatologist performed a biopsy of the left lower abdomen, revealing a diagnosis of MF. At MDACC, triamcinolone 0.1% cream was prescribed for the patient, and he received UVB light therapy three times a week with noticeable improvement of the lesions. A full-body computed tomography (CT) scan was performed, which revealed an 8 centimeter (cm) x 5.6 cm mass in the upper pole of the right kidney. Urinary testing was positive for hematuria. The patient denied any urinary symptoms. Flow cytometry was negative for atypical T cells in peripheral blood. A biopsy of the mass revealed clear cell RCC. The patient underwent a laparoscopic right radical nephrectomy in October 2003 to remove the mass. Follow-up CT scan in March 2004 demonstrated no RCC recurrence and complete remission of MF. The patient still remains disease-free from RCC and MF.

### 2.2. Case 2: Patient B

**Figure 1:**
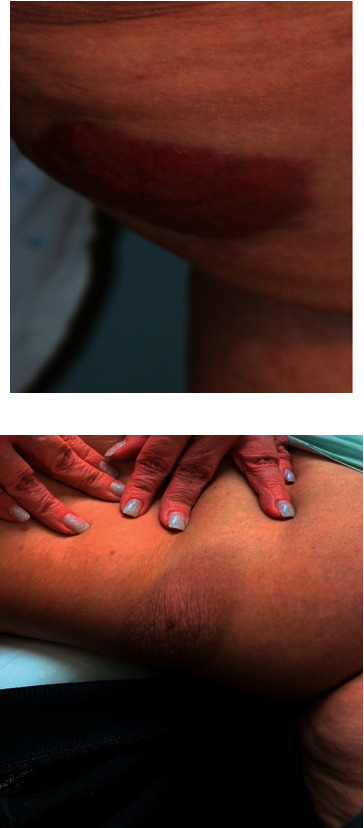
Patient B: clinical images of MF tumor before (a) and 1 month after (b) the removal of RCC and local radiation therapy.

A 52-year-old female presented to MDACC in October 2019 for the management of MF with large cell transformation. In June 2019, she noticed a lesion on her left thigh resembling a spider bite and multiple “eczema-like” patches on the forearms and feet. Her local dermatologist performed a punch biopsy on the left lateral back and left posterior thigh, revealing MF with large cell transformation. Flow cytometry was negative for atypical T cells in peripheral blood. Triamcinolone 0.1% cream was prescribed for the patient. At MDACC, full-body skin exam revealed a 10.5 cm × 6.5 cm tumor on the left inner thigh (Figure 1) and a 3 cm × 4 cm tumor on the right posterior thigh. A positron emission tomography/computed tomography (PET/CT) scan revealed enlarged FDG-avid bilateral inguinal and right external iliac lymph nodes and an 8.5 cm × 7.7 cm exophytic right renal mass with extension into the renal sinus. Lymph node biopsy of the left inguinal lymph node demonstrated evidence of CD4-positive T-cell lymphoma. A biopsy of the mass revealed clear cell RCC. The patient underwent an open right radical nephrectomy and regional lymphadenectomy in December 2019. She received local radiation therapy to the lesions and one cycle of romidepsin. Follow-up CT scan demonstrated complete response of her lymph nodes and near complete response of her skin lesions. She remains stable on observation.

### 2.3. Case 3: Patient C

**Figure 2:**
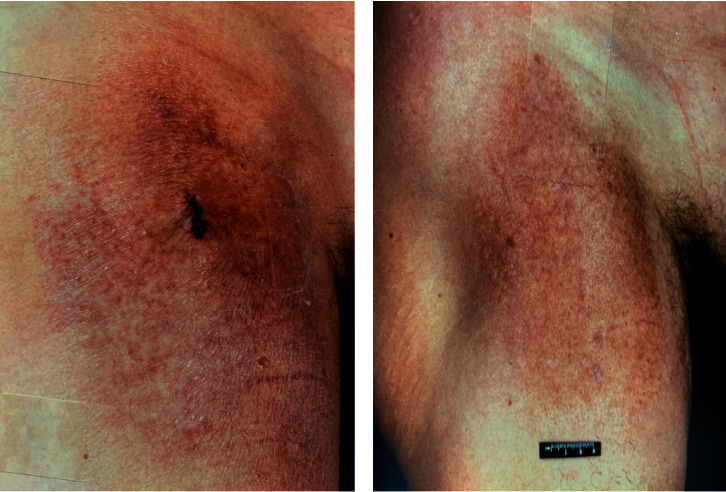
Patient C: clinical images of MF lesion before removal of RCC (a) and 2 years following removal of RCC (b).

A 66-year-old male presented to MDACC in July 1996 for the management of stage IA MF. The patient first noticed a lesion on his right thigh approximately 10 years prior. He was diagnosed as having “dry skin” and treated with an unknown ointment with no improvement. The lesion continued to expand, becoming raised and indurated. In June 1996, a skin biopsy was performed by the patient's local dermatologist, which was consistent with MF. On presentation at MDACC, full-body skin exam revealed an 18 cm × 13 cm dusky red plaque on the right upper thigh with a net-like pattern. The plaque was rebiopsied at MDACC, which confirmed MF. The patient's MF lesion was treated with tazarotene 0.05% gel and mometasone 0.1% cream with minimal improvement. In February 1998, the patient was hospitalized for atrial fibrillation. During hospitalization, he was found to have a 3.3 cm × 2.5 cm solid mass on the left lower pole of the kidney. A biopsy of the mass revealed clear cell RCC. The patient denied any urinary symptoms. A left partial nephrectomy was performed. Within months of removal of the renal mass, the patient began to notice significant flattening and decreased hyperpigmentation of his MF lesion without changes to his treatment regimen (Figure 2). His skin lesion has since remained stable.

## 3. Discussion

Decreased cellular immunity and immune surveillance in MF patients may be a driving factor for secondary malignancies [2]. While the occurrence of RCC in our patients may have been incidental, the expanded number of CD4+ lymphocytes present in existing MF may have triggered the development of RCC. Using both in vitro and in vivo studies of mice RCC tissue, Wang et al. found that RCC cells have a better ability to recruit CD4+ lymphocytes than normal renal cells [5]. The recruited CD4+ lymphocytes stimulate RCC cell proliferation, promoting RCC [5].

The senior authors of this manuscript report that, in their clinical experience, nearly all MF patients have a tendency to relapse despite adequate treatment, and periodic flares are expected throughout their lifetime. In our patients, the absence of MF flares in over 16 years following removal of their RCC increases suspicion of a paraneoplastic syndrome. Paraneoplastic syndrome refers to specific clinical manifestations that are triggered by an altered immune system in response to a malignancy; the symptoms may not be directly attributable to invasion of the primary tumor, metastasis, or direct compression [6]. The stimulation of lymphocytes in response to RCC may have triggered the development of MF, and subsequent removal of the RCC resulted in return to normal lymphocyte levels, halting progression of MF.

While RCC was found after the diagnosis of MF in each of these patients, it remains unknown how long the RCC was present. Due to the lack of widely accepted guidelines regarding screening for renal cancer, more stringent screening guidelines may be required. Routine screening for underlying solid organ malignancies in patients with MF may be costly and impractical. However, screening is warranted in patients presenting with symptoms that may indicate an underlying malignancy, and greater emphasis should be placed on age-appropriate cancer screenings in MF patients. Refractory or rapidly progressing disease may require CT scan.

## 4. Conclusion

Patients with MF are at higher risk for second malignancies [4]. Of the malignancies that have been associated with MF, renal cell carcinoma (RCC) and other solid tumor malignancies have not been studied extensively [2]. The presence of a second malignancy, such as RCC, in MF patients may be a result of a paraneoplastic syndrome. Further research is needed to characterize the association between MF and RCC.

## Data Availability

No data were used to support this study.

## References

[1] Amber K. T., Bloom R., Nouri K. (2016). Second primary malignancies in CTCL patients from 1992 to 2011: a SEER-based, population-based study evaluating time from CTCL diagnosis, age, sex, stage, and CD30+ subtype. *American Journal of Clinical Dermatology*.

[2] Goyal A., O’Leary D., Goyal K. (2020). Increased risk of second primary hematologic and solid malignancies in patients with mycosis fungoides: a Surveillance, Epidemiology, and End Results analysis. *Journal of the American Academy of Dermatology*.

[3] Kim Y. J., Shin H. J., Won C. H. (2018). The incidence of other primary cancers in patients with cutaneous lymphoma. *Annals of Dermatology*.

[4] Brownell I., Etzel C. J., Yang D. J., Taylor S. H., Duvic M. (2008). Increased malignancy risk in the cutaneous T-cell lymphoma patient population. *Clinical Lymphoma and Myeloma*.

[5] Wang Y., Wang Y., Xu L. (2018). CD4+ T cells promote renal cell carcinoma proliferation via modulating YBX1. *Experimental Cell Research*.

[6] Dimitriadis G. K., Angelousi A., Weickert M. O., Randeva H. S., Kaltsas G., Grossman A. (2017). Paraneoplastic endocrine syndromes. *Endocrine-Related Cancer*.

